# A Surveillance of Paracetamol and Nonsteroidal Anti-Inflammatory Drug Consumption in Cluj-Napoca, Romania, Using Wastewater-Based Epidemiology

**DOI:** 10.3390/metabo15090576

**Published:** 2025-08-28

**Authors:** Mihail Simion Beldean-Galea, Mihaela-Cătălina Herghelegiu, Audrey Combès, Jérôme Vial, Robert Tötös, Maria Concetta Bruzzoniti, Maria-Virginia Coman

**Affiliations:** 1 Faculty of Environmental Science and Engineering, Babeş-Bolyai University, 30 Fântânele Str. 30, 400294 Cluj-Napoca, Romania; 2 Raluca Ripan Institute for Research in Chemistry, Babeş-Bolyai University, 30 Fântânele Str. 30, 400294 Cluj-Napoca, Romania; 3 École Supérieure de Physique et de Chimie Industrielle de la Ville de Paris, Paris Sciences et Lettres University, 10 Rue Vauquelin, Cedex 05, 75231 Paris, France; 4 Faculty of Chemistry and Chemical Engineering, Babeş-Bolyai University, Árány Janos Str. 11, 400028 Cluj-Napoca, Romania; 5 Department of Chemistry, University of Turin, Via P. Giuria 5, 10125 Turin, Italy; mariaconcetta.bruzzoniti@unito.it

**Keywords:** paracetamol, nonsteroidal anti-inflammatory drugs, biomarkers, wastewater-based epidemiology, human consumption

## Abstract

Paracetamol and nonsteroidal anti-inflammatory drugs are the most popular first-line analgesics, being freely available without any medical prescription. For this reason, it is difficult to estimate their actual consumption among the population. One tool for surveillance of pharmaceutical use is wastewater-based epidemiology, a useful approach for monitoring public health by analyzing specific biomarkers in wastewater. **Background/Objectives**: In this study, the consumption of paracetamol and four nonsteroidal anti-inflammatory drugs (ibuprofen, naproxen, ketoprofen, and diclofenac) was evaluated by analyzing their residues as specific biomarkers in wastewater and the fraction excreted as drug metabolites in urine. **Methods**: For this purpose, composite wastewater samples were collected from the influent of the wastewater treatment plant in Cluj-Napoca, Romania, in four sampling campaigns (September 2021, February 2022, February 2024, and October 2024), and the target biomarkers were analyzed by liquid chromatography–tandem mass spectrometry. **Results**: The results of consumption expressed in g/day/1000 inhabitants showed variations for the five studied pharmaceuticals in the following ranges: 6.65–185.57 for paracetamol, 0.32–2.44 for ibuprofen, 0.29–0.82 for naproxen, 0.21–2.65 for ketoprofen, and 0.23–1.11 for diclofenac, depending on the sampling period. This variation can be explained either by the different behaviors regarding the consumption of the pharmaceutical products studied by the population during the sampling periods or by an inappropriate estimate of the number of inhabitants connected to the sewage system. **Conclusions**: Future studies need to establish a more comprehensive model that considers many other variables that may influence the results obtained through WBE.

## 1. Introduction

The presence of pharmaceutical products in wastewater is a well-known fact due to their intensive use in human and veterinary treatments, respectively, and their incorrect disposal [1,2,3,4]. Starting from the premise that everything that is consumed ends up in the sewer, it is possible to estimate the consumption of a certain drug by the population either through back-calculation or using the information related to the drug as such or a specific biomarker (metabolite product) [5,6,7,8]. A method to obtain qualitative and quantitative information about lifestyle, exposure, or ingestion of a certain type of drug, without knowing the identity of individuals, is wastewater-based epidemiology (WBE), also called “sewage epidemiology” or “sewage based epidemiology” [9,10]. The advantages of this methodology are that it provides objective and almost real-time data, monitoring different consumption rates in a group of individuals and not just one, respecting privacy [9,10]. The disadvantages are generated by the lack of information on the purity, dosage, or frequency of use of the compound of interest and, in some cases, the mode of administration, a single compound, or concomitantly with other compounds. As a result, the method does not clearly reflect the number of consumers because the amount consumed is normalized (mg/d/1000 inh or doses/d/1000 inh), does not distinguish between high consumption due to an increase in the number of consumers or an increase in the dose consumed, and cannot estimate the price of the compound of interest [11,12,13]. Other methods by which drug consumption can be assessed are the centralization of medical prescriptions, market surveys, questionnaires, and hospital and police reports, but these only cover a limited population, are time-consuming, sometimes cost-effective, and are not carried out in real time [7,8,11].

Starting from the fact that wastewater represents a complex matrix and can be seen as a mirror of society, which can offer new perspectives on different drug consumption [10,14], at first, WBE was used to estimate illicit drug use [6,15,16]. Later, it was extended to estimate consumption of tobacco [17], caffeine [18], alcohol [19], pharmaceuticals, and personal care products [20,21,22,23,24] and, recently, in the surveillance of the Coronavirus Disease 2019 (COVID-19) [25,26,27].

Although WBE is an effective method used to reveal the consumption of different pharmaceutical compounds [28,29], there are still uncertainties regarding estimating population size [30], pharmacokinetic data [31,32], and stability of pharmaceuticals in sewage [33,34].

The estimation of the population size served by the wastewater catchment can be performed using census data, hydrochemical parameters in wastewater (chemical oxygen demand (COD), biological oxygen demand (BOD), total nitrogen (N), nitrogen ammonia (NH_4_-N), or total Phosphorous (P)) [35,36], or design capacity [30]. Thomas et al. [37] proposed the use of mobile device data to estimate the dynamics of population size, and, recently, the population estimated based on mobile phone data was approximately the same as that based on census data [38]. However, these approaches are inappropriate, as population census data are updated every 10 years and do not take into account real-time population movement [30], while hydrochemical quality parameters may be influenced by industrial, domestic, or mixed discharges [11,30,36,39]. Other endogenous markers like creatinine were proposed to be used for the quantification of the population, but it has low stability, and no correlation was found between daily loads of creatinine and population [40]. Cholesterol coprostanol is excreted via feces and presents affinity to particulate matter, while cotinine and 5-hydroxyindoleacetic acid (5-HIAA) showed good linearity between excretion and census population [41]. The most appropriate estimation was provided by Been et al. [36], which showed that NH_4_-N is a suitable marker to monitor population variation in real time, being formed mainly from urinary excretion and unaffected by non-human activities.

During the global Severe Acute Respiratory Syndrome Coronavirus 2 (SARS-CoV-2) pandemic (2019–2022), self-medication, lack of specific therapeutic treatments, and exaggerated practices led to overuse of pharmaceuticals and ultimately to increased mass loading of pharmaceuticals in wastewater. The European Medicines Agency (EMA) has advised the use of nonsteroidal anti-inflammatory drugs (NSAIDs) such as ibuprofen and paracetamol to treat SARS-CoV-2, taking into account product information recommendations and the lowest effective dose for a period as short as possible [42].

Paracetamol is widely used to treat minor pain, headache, and fever, and due to its relative lack of adverse side effects, it is considered first-line therapy [43], having limited anti-inflammatory effects [44]. NSAIDs, such as ketoprofen, naproxen, ibuprofen, or diclofenac, are traditionally used to relieve various symptoms of fever, inflammation, migraine, osteoarthritis, and rheumatoid arthritis, and their side effects are abdominal pain, bleeding, and kidney failure [22,45].

In 2019, the global analgesics (paracetamol (acetaminophen), nonsteroidal anti-inflammatory drugs, and opioid drugs) market size reached 48.2 billion USD; in 2022, it reached 51.8 billion USD and is expected to reach 67.4 billion USD by 2028 [46]. In Romania, in 2019, the pharmaceutical industry manufactured approximately 10 million boxes of paracetamol (200 million tablets) and 1 million boxes of ibuprofen (10 million tablets), these two main drugs also being the most used in the treatment of symptoms caused by SARS-CoV-2 [47].

Starting from the premise that WBE demonstrated a wide range of applications mentioned above, the current study aims to provide additional information related to the consumption of paracetamol and NSAIDs among the population using the residues of these pharmaceuticals in wastewater as biomarkers. For this purpose, four different sampling campaigns in Cluj-Napoca, Romania, were carried out, two campaigns during the peak periods of the SARS-CoV-2 pandemic (September 2021 and February 2022) and two campaigns after the pandemic (February 2024 and October 2024), to observe differences in the human consumption of these drugs among the population.

The concentrations of paracetamol and selected NSAIDs were determined from wastewater samples, collected from the influent of the Cluj-Napoca wastewater treatment plant, by liquid chromatography–tandem mass spectrometry (LC-MS/MS). Finally, the concentrations of the pharmaceuticals were converted to consumption calculations (g/d/1000 inh), taking into account the mean flow of the influent of WWTP, the correction for compound-specific pharmacokinetics, and the size of the contributing population [7,9,22,48].

It is important to note that this work represents the first WBE study conducted in Romania, which aims to study the link between the consumption of paracetamol and NSAIDs (ketoprofen, naproxen, ibuprofen, and diclofenac) and the state of public health.

## 2. Materials and Methods

### 2.1. Chemicals and Reagents

The reference substances of the studied pharmaceuticals (paracetamol (PARA), ibuprofen (IBU), ketoprofen (KET), naproxen (NAP), and diclofenac sodium salt (DIC)) were purchased from Sigma-Aldrich (Steinheim, Germany). Stock standard solutions of each individual pharmaceutical at 1000 mg/L concentration were prepared in acetonitrile, except diclofenac, which was prepared in methanol. All solutions were stored at 4 °C in the dark until their use. The molecular structure and some physicochemical properties of the studied pharmaceuticals are given in Supplementary Materials (Table S1).

Liquid chromatography grade methanol (MeOH) and acetonitrile (ACN), formic acid (FA), and hydrochloric acid were acquired from Merck (Darmstadt, Germany). Ultrapure water was prepared using a Milli-Q ultrapure system, MF-Millipore™ Membrane Filter, 0.45 µm pore size, hydrophilic PVDF for filtration (Millipore, MA, USA). Strata X cartridges 200 mg/3 mL were purchased from Phenomenex (Torrance, CA, USA).

### 2.2. Instrumentation

The analyses were performed using a liquid chromatograph (UltiMate 3000, Thermo Scientific, Waltham, MA, USA) coupled with a triple quadrupole mass spectrometer (TSQ Quantum, Thermo Scientific) equipped with electrospray ionization source (ESI).

For paracetamol, the chromatographic separation was performed on a ZIC-HILIC column (SeQuant, 150 × 2.1 mm, 3.5 µm) heated at 40 °C in isocratic mode with a mobile phase composed of ACN:H_2_O:FA (94.93:5:0.07, *v*/*v*/*v*). The flow rate was set at 0.2 mL/min, the injection volume at 5 µL, and the run time at 22 min. The mass spectrometer was operated in the positive mode with multiple reaction monitoring detection using an electrospray voltage of 3000 V, and the sheath gas and ion sweep gas pressures were zero. The collision gas was argon at a pressure of 1.5 mTorr. Capillary and vaporizer temperatures were set at 260 °C and 280 °C, respectively, and tube lens offset of 60 V was used.

For NSAIDs separation, a Supelco Ascentis Express RP-Amide column (100 × 3.0 mm, 5 µm), heated at 40 °C, was used. A gradient elution mode was applied using a mobile phase composed of (A) H_2_O with FA (0.1%):(B) MeOH. The gradient was as follows: starting at 0% B for 0.5 min, increasing to 40% B in 2.5 min, followed by an increase to 70% B in 4.5 min and then to 95% B in 1.5 min and held at 95% B for 2 min, then to 100% B in 1 min and held for 10 min. The flow rate was set to 0.3 mL/min, the injection volume to 5 µL, and the run time to 22 min. The mass spectrometer was operated in negative mode with multiple reaction monitoring detection using an electrospray voltage of 4000 V, and the sheath gas and ion sweep gas pressures were 20 and 2.0, respectively. The collision gas was argon at a pressure of 1.5 mTorr. Capillary and vaporizer temperatures were set at 300 and 260 °C, respectively, and tube lens offset of –80 V was used.

MS/MS transitions were acquired for each compound using the most sensitive ions as precursors and product ions (Table S2). For the linearity studies, calibration solutions were prepared in incremental amounts between 10 ng/mL and 1000 ng/mL for PARA, between 50 ng/mL and 1500 ng/mL for NAP, IBU, DIC, and between 100 ng/mL and 2000 ng/mL for KET. Five-point calibration curves were generated as linear functions with a correlation coefficient (R^2^) ≥ 0.96. The limit of detection (LOD) and limit of quantitation (LOQ) were calculated by considering the standard deviation of the response factor of the detector (σ) for each compound and the slope (S) of each calibration curve, according to the following equations: LOD = 3.3 × σ/S and LOQ = 10 × σ/S, respectively [49].

The intraday and interday precision of the LC-MS/MS method, expressed as relative standard deviation, were obtained by repeated measurements of the standard mixture of 100 ng/mL of PARA, NAP, DIC, and IBU, respectively, and 200 ng/mL for KET. The intraday precision (n = 6 series) was obtained on the same day (RSD ≤ 14.05%), while the interday precision (n = 3 series/day) was obtained on three consecutive days (RSD ≤ 7.58%). The obtained values show a good intraday and interday precision of the developed method.

The preconcentration factor of the method (1000) was calculated based on the ratio between the initial volume of the aqueous sample (50 mL) and the final volume obtained (50 μL) subjected to analysis, and it was used to determine the method detection and quantification limits (LDM, LQM), which were also low (ng/L).

The performance of the validated LC-MS/MS methods is presented in Table S2.

### 2.3. Sample Preparation

Sample preparations for PARA and NSAIDs determination were reported in our previous work [50] with minor modifications necessary for analysis by liquid chromatography–tandem mass spectrometry.

Before extraction, wastewater samples were defrosted and filtered with 0.45 µm membrane filters. Each sample was divided into two parts: One was spiked before extraction with 10 ng of paracetamol and 100 ng of NSAIDs, and the second one was spiked with the same amount after the extraction. The selected pharmaceuticals were extracted from the wastewater samples using a Strata X cartridge. The SPE cartridges were preconditioned with 6 mL of Milli-Q water and 6 mL of methanol and again with 6 mL of Milli-Q water. Then, water samples were passed through the cartridges at a flow rate of approximately 1 mL/min. Subsequently, the cartridges were dried for 20 min under vacuum. Finally, the retained compounds were eluted with 4 mL of methanol. The extract was evaporated to dryness under a gentle stream of nitrogen, and the residue was dissolved in 50 µL acetonitrile for LC-MS/MS analysis.

Recoveries were determined by spiking wastewater with target compounds (10 ng and 100 ng) following the same pretreatment procedures. The differences in responses between spiked and non-spiked wastewater extracts were used to assess the matrix effect [51].

### 2.4. Sample Collection

The composite wastewater samples were collected from the influent of the wastewater treatment plant (WWTP) that receives wastewater from around 400,000 people from the city of Cluj-Napoca (300,000 inhabitants) and the communes of Florești (55,000 inhabitants), Baciu (14,000 inhabitants), Gilău (9000 inhabitants), and Săvădisla (5000 inhabitants) [52].

Wastewater sampling was performed hourly, applying a time-proportional sampling mode, using the automatic sampling device located at the inlet of the wastewater treatment plant. After sampling, the individual hourly samples collected over 24 h were mixed to obtain representative daily composite samples. Each composite sample was collected in a clean polypropylene flask, kept at 4 °C in the dark, transported to the laboratory, and stored in a freezer until analysis.

The number of composite samples collected in four campaigns was nine for September 2021 and February 2022 and seven for February 2024 and October 2024. The number of inhabitants served by the WWTP of Cluj-Napoca, as well as the pharmacokinetic data for the detected compounds and the daily flow rates, were used to back-calculate the consumption of the pharmaceutical compounds.

### 2.5. Consumption Estimation Through WBE

Measured concentrations of PARA and NSAIDs (expressed in ng/L) were multiplied by daily wastewater flow rates (m^3^/day) recorded at WWTP and divided by the number of inhabitants to obtain the mass loads (Equation (1)). The consumption of PARA and NSAIDs was calculated by multiplying the fraction excreted as pharmaceutical metabolite in urine (%) by the ratio of the molecular mass of the parent pharmaceutical to the molecular mass of the pharmaceutical metabolite (Equation (2)) [6,7,22,48]. In our study, each pharmaceutical is used as a biomarker of its own consumption.

(1) $$Massload(mg/d/1000\;inh)=\frac{C_{i}\left(\frac{ng}{L}\right)\times Q_{in}\left(\frac{m^{3}}{d}\right)\times 10^{-3}}{\frac{P}{1000}}$$

(2) $$Consumption(mg/d/1000\;inh)=Load\times \frac{1}{{EF}_{i}}\times \frac{{MW}_{par}}{{MW}_{met}}$$

where *C_i_* is the influent concentration of a pharmaceutical; *P* is the number of inhabitants served by the WWTP; *Q_in_* is the mean influent flow of the WWTP; *MW_par_* is the molecular mass of the parent pharmaceutical; *MW_met_* is the molecular mass of the metabolite pharmaceutical; *EF_i_* is the fraction of a given pharmaceutical excreted as the metabolite through urine.

## 3. Results

### 3.1. Estimating Population Size

In the present study, the number of people served by the wastewater treatment plant was estimated using the hydrochemical parameters provided by the WWTP from Cluj-Napoca. Thus, the number of inhabitants was established by dividing BOD by 60, COD by 128, NH_4_-N by 8.5, and P by 1.7 [30,36,39] as estimations of the amount produced by individuals and multiplied by the flow rates.

COD was measured every day and showed a large variation, especially in September when a music festival took place, due to the influx of people and light rain (300,968 inh), while in February, the variation was small (280,908 inh). During the February 2024 sampling campaign, there was only one rainy day (13 February 2024), in which an increase in the value of COD and implicitly the number of inhabitants is clearly observed (636.00 mg/L, i.e., 469,225 inh); the average number of inhabitants estimated based on this parameter is 281,426 inh. In the October 2024 sampling campaign, there was no precipitation and no high population influx; the only additional people present were students, and the average population estimated based on COD is 286,091 inh.

BOD was monitored two times in the September 2021, February 2022, and February 2024 sampling campaigns and three times in October 2024. The population estimated using BOD was generally lower compared to that estimated using COD in September 2021 (250,582 inh) and in February 2022 (186,881 inh), except in February 2024 (301,862 inh) and October 2024 (345,931 inh), when it was higher than that estimated using COD.

P and NH_4_-N were measured once per campaign. The estimated population for the sampling campaign in September 2021, based on P, is 294,532 inh, and based on NH_4_-N, it is 524,394 inh. For February 2022, the estimated population based on P is 172,644 inh, and for NH_4_-N, it is 498,718 inh [53]. For February 2024, the estimated population based on P is 334,348 inh, and based on NH_4_-N, it is 439,233 inh, while for October 2024, the estimated population based on P is 421,666 inh, and for NH_4_-N, it is 532,261 inh (Table S3).

The population estimated based on P is lower compared to the other hydrochemical parameters in all sampling campaigns and does not correlate with the estimated values of inhabitants using census data. These results indicate that COD, BOD, and P are difficult to apply for real-time estimation of the population size [30]; the only one that can be taken into consideration is NH_4_-N [54], but uncertainties in population estimations can be made, especially in the wet season [36].

For further calculations, we considered the estimated number of inhabitants based on NH_4_-N, which correlates better with census data. Thus, the estimated number of inhabitants is 524,394 inh for September 2021, 498,718 inh for February 2022, 439,233 inh for February 2024, and 532,261 inh for October 2024.

Hydrochemical parameters and values of the inhabitants for the September 2021 and February 2022 sampling campaigns can be seen in [53], and those for the February 2024 and October 2024 sampling campaigns are available in Table S3.

### 3.2. Occurrence of PARA and NSAIDs in the Influent of Cluj-Napoca WWTP

The values measured in all samples analyzed in the four monitoring campaigns show an occurrence of the selected pharmaceuticals in all samples (frequency of detection 100%). The measured values vary from subunit concentrations to tens of μg/L (Table 1).

The largest variation was recorded for PARA with a minimum value of 0.88 μg/L in February 2022 and a maximum value of 24.83 μg/L in September 2021. KET values varied between 0.75 μg/L in February 2022 and 9.34 μg/L in September 2021. The minimum value for NAP of 1.28 μg/L was recorded in September 2021 and the maximum of 3.75 μg/L in October 2024. IBU concentrations varied between 1.33 μg/L in February 2022 and 11.80 μg/L in October 2024. For DIC, the minimum concentration of 0.97 μg/L was recorded in September 2021 and the maximum of 4.73 μg/L in February 2024. The values measured for each campaign and composite sample analyzed, respectively, and the average values are given in the Supplementary Materials (Table S4).

**Figure 1:**
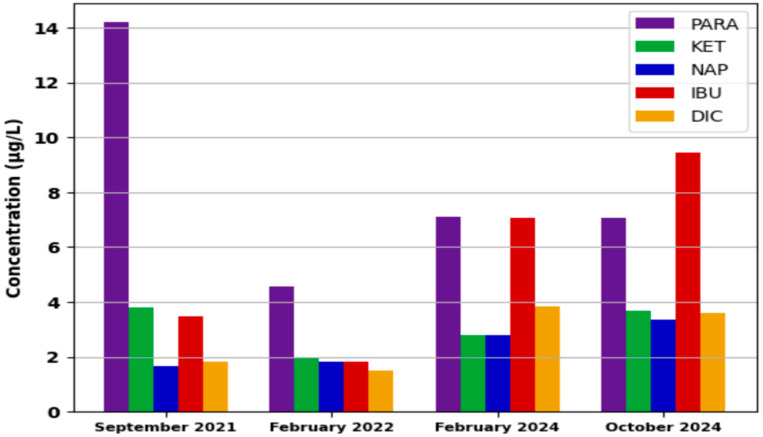
Average concentration of PARA and NSAIDs registered in the monitoring campaigns.

In order to better observe the differences in concentration, respectively, and the trend recorded in the four monitoring campaigns, the average concentrations were calculated for each campaign. In Figure 1, the differences in average concentrations among the four monitoring campaigns are presented.

As can be seen in Figure 1, the average concentration recorded during the four monitoring campaigns changes significantly. The high average concentrations recorded for PARA in the September 2021 campaign can be attributed to the peak of infection corresponding to the COVID-19 pandemic [55], but this hypothesis cannot be correlated with the number of cases of COVID-19 infection since there was also a peak of infection in February 2022, but consumption was low. In the 2024 campaigns, the average concentrations of PARA were quite similar (7.06 and 7.09 µg/L) between the values in 2021 and 2022, respectively.

In the case of NSAIDs, an increase in the concentration is observed, especially in campaigns from 2024. For February 2024, this increase can be attributed to the respiratory infection season in Romania but also to a period without precipitation, the wastewater inflow into the treatment plant [Table S3] being much lower compared to September 2021 and February 2022 [53]. The higher average concentration of NSAIDs recorded in October 2024 compared to February 2024 can be attributed to the beginning of respiratory infections that usually occur after school holidays, respectively, to the increase in the number of inhabitants in the city due to the start of the academic school year (Table S3).

However, these concentrations are reported per liter of wastewater and do not provide information regarding the consumption of these drugs among the population. Other approaches are necessary to find out the consumption.

### 3.3. Estimated PARA and NSAIDs Consumption by WBE

Consumption levels of PARA and NSAIDs were estimated by using Equations (1) and (2). For each parent compound, the estimated population using the hydrochemical parameters connected to the wastewater treatment plant was used to calculate the consumption levels of PARA and NSAIDs. A correction factor obtained by multiplying the urinary excretion rate of the pharmaceutical by the ratio of the molecular mass of the parent compound to the metabolite was also used. As biomarkers, in this study, the parent compounds were considered as products of human metabolism, and we assumed that their origin was only human consumption. Thus, for the pharmaceuticals of interest, the excretion rates considered are the following: paracetamol 3%, ketoprofen 80%, and naproxen, ibuprofen, and diclofenac 95% [22,28,32,56].

The results of our study show a wide variation in the consumption of selected drugs, depending on the monitoring period. The highest variation in consumption was recorded for PARA, while for NSAIDs, the variation is lower. The minimum, maximum, and average values calculated for each monitoring campaign are presented in Table 2.

As can be seen in Table 2, the highest variation in consumption was recorded for PARA, with a minimum value of 6.65 g/d/1000 inh recorded in February 2022 and a maximum value of 185.57 g/d/1000 inh in September 2021. KET consumption values varied between 0.21 g/d/1000 inh in February 2022 and 2.65 g/d/1000 inh in September 2021. For NAP, the minimum consumption of 0.29 g/d/1000 inh was recorded in September 2021, while the highest value of 0.82 g/d/1000 inh was in February 2024. The minimum consumption value for IBU of 0.32 g/d/1000 inh was recorded in February 2022 and the maximum of 2.44 g/d/1000 inh in October 2024. DIC recorded a minimum consumption of 0.23 g/d/1000 inh in September 2021, and a maximum consumption of 1.11 g/d/1000 inh in February 2024. The consumption values calculated for each day of the monitoring campaign, respectively, and the average values, are presented in the Supplementary Materials (Table S5).

**Figure 2:**
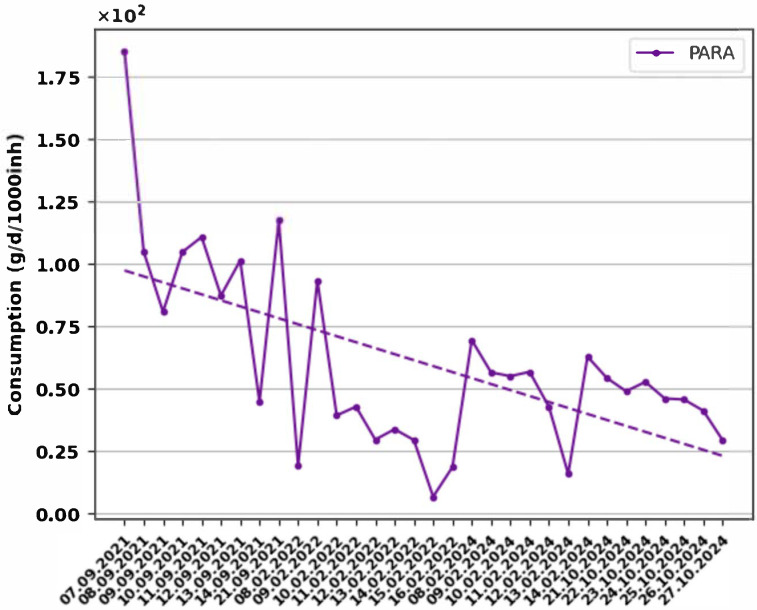
Consumption trend of PARA registered in the monitoring campaigns.

**Figure 3:**
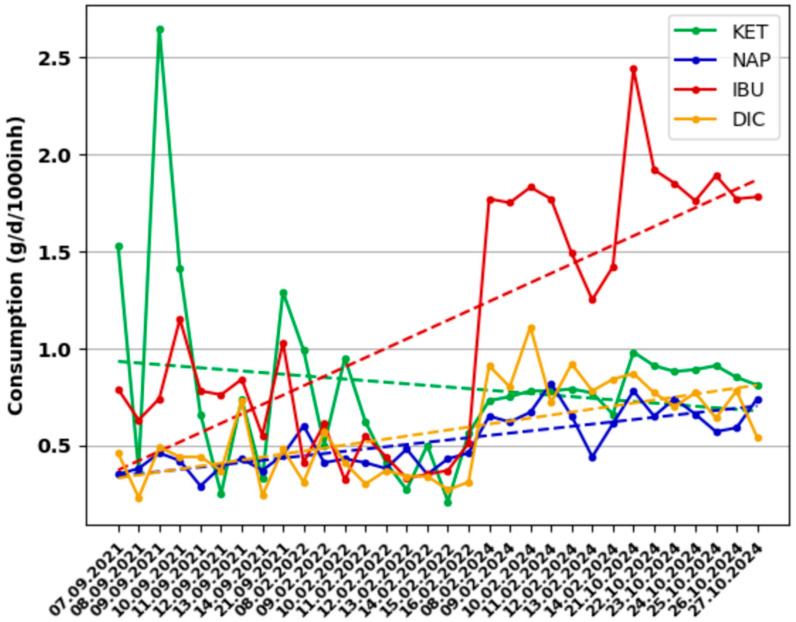
NSAIDs consumption trend recorded in monitoring campaigns.

The population’s behavior regarding the consumption of certain pharmaceutical products was assessed by tracking the consumption trend during the monitoring period (Figure 2 and Figure 3).

Analyzing Figure 2, it can be seen that in the case of PARA, the consumption trend is decreasing from September 2021 to October 2024. The high consumption in September 2021 may be due to the significant influx of people attending a music festival but also to the light rainfall in the first days of the sampling campaign. It should be noted that there is no separate rainwater collection system in the city of Cluj-Napoca, and social and meteorological events may influence the presence of the studied compounds in wastewater [57].

In the case of NSAIDs (Figure 3), for KET, there is a decreasing trend in consumption between September 2021 and February 2022, respectively, and an increasing trend starting with February 2024. In the case of NAP and DIC, an increasing trend is observed throughout the monitoring period, while in the case of IBU, after a slight decrease in consumption in 2022, a significant increasing trend in consumption is recorded in 2024.

Even though the results of this study contain a series of errors related to the estimation of the number of individuals connected to the sewage system, the increasing trend in consumption is evident. This is also proven by statistical data on the production of medicines in Romania, which has increased significantly since 2019 [47].

The same growth trend is also being recorded at the global level. The market size for PARA is currently estimated to reach approximately USD 16.8 billion by 2034, up from USD 10.8 billion in 2024 [58], and the market size for NSAIDs is estimated to reach USD 31.29 billion by 2030 [59], up from USD 22.58 billion in 2024. This requires the establishment of long-term drug monitoring policies and health education on the consequences of drug abuse.

## 4. Discussion

### 4.1. Comparison with Other Studies

Using the results obtained through the WBE approach, a comparison was made between the results obtained in this study for the selected pharmaceuticals and other results obtained worldwide (Table 3).

The estimated consumption of PARA (6.65–185.57 g/d/1000 inh) in this study is lower than that in Vietnam (283.00 ± 0.05–473.00 ± 131.00 g/d/1000 inh) [60] and China (0.0073–250.00 g/d/1000 inh) [63] but higher than that in Austria (1.80–15.20 g/d/1000 inh) [14], the United Kingdom (28.09 ± 11.04–61.29 ± 48.13 g/d/1000 inh) [32,65], South Africa (0.79–21.37 g/d/1000 inh) [61], Greece (19.01–56.61 g/d/1000 inh) [28], the second available study from China (0.03–0.84 g/d/1000 inh) [62], and Spain (5.91–17.99 g/d/1000 inh) [64].

For KET, the estimated consumption (0.21–2.65 g/d/1000 inh) in this study is slightly higher than that in the United Kingdom (0.12–0.15 g/d/1000 inh) [65] and Greece (0.11–0.28 g/d/1000 inh) [28].

Regarding NAP, the estimated consumption (0.29–0.60 g/d/1000 inh) in this study is much lower than that in Greece (10.34–10.97 g/d/1000 inh) [28] and the United Kingdom (3.56 ±2.67–4.16 ± 3.9 g/d/1000 inh) [32] but consistent with that in South Africa (0.04–0.71 g/d/1000 inh) [61] and China (0.0002–0.51 g/d/1000in) [62].

The estimated consumption obtained in this study for IBU (0.32–1.15 g/d/1000 inh) is consistent with that in Greece (0.75–1.88 g/d/1000 inh) [28] but lower than in Vietnam (3.60 ± 0.60–4.60 ± 0.50 g/d/1000 inh) [60] and the United Kingdom (3.29 ± 2.16–5.58 ± 1.96 g/d/1000 inh) [65] and much lower than that in China (0.58–321.00 g/d/1000 inh) [63].

The results of this study for estimated DIC consumption (0.23–1.11 g/d/1000 inh) are slightly higher but in the same range of magnitude as those obtained in the United Kingdom (0.10 ± 0.05–0.20 ± 0.10 g/d/1000 inh) [65], South Africa (0.02–0.27 g/d/1000 inh) [61], Greece (0.63–1.00 g/d/1000 inh) [28], and China (0.004–0.23 g/d/1000 inh) [62].

From the above, it can be concluded that there are no major differences in the consumption behavior of the selected pharmaceuticals, which vary in the same size ranges in different regions of the world. The small differences that appear can be attributed to errors that are associated with the WBE methodology, which are accepted and recognized by the scientific literature.

### 4.2. Uncertainties

Our study estimated the quantities of PARA and NSAIDs consumed during two peak periods of SARS-CoV-2 infections and two periods of seasonal respiratory infections in Cluj-Napoca, Romania, and we acknowledge a few uncertainties.

First, as in many other countries in the world, in Romania, the fear of contracting SARS-CoV-2 and the lack of a specific treatment led people to stock up on various pharmaceutical products (antibiotics, antivirals, nonsteroidal anti-inflammatory drugs, and especially paracetamol) as a precautionary measure. Given the large quantities of pharmaceuticals purchased and the lack of a specific management program for expired pharmaceuticals, we can assume that a large part of the purchased pharmaceuticals ended up in the sewage system, either due to consumption or due to the expiration of the validity period, thus influencing the estimated consumption. It is estimated that in Romania, approximately 1500 tons of expired medicines end up in the sewer or garbage annually [66], and from the garbage, they end up in the landfill and the landfill leachate in the WWTP.

Second, all pharmaceuticals we investigated are also used in veterinary practices [67,68], and the excretion profile of humans and animals may be different. Thus, if there were wastewater discharges from animal husbandry to the WWTP, the consumption estimate would be affected.

Third, the pattern of pharmaceutical use makes the back-calculation of consumption more complicated. The use of PARA and NSAIDs as a biomarker of self-consumption is likely to lead to overestimation due to topical applications, and the use of metabolites (glucuronides) will lead to an underestimation of consumption since topical use of NSAIDs is not taken into account. A combined approach is recommended for estimating total use [32].

And last but not least, the estimation of population size using hydrochemical parameters can be influenced by events, industrial and domestic discharges, or weather.

## 5. Conclusions

This study represents the first attempt in Romania to estimate the consumption of PARA and NSAIDs (KET, NAP, IBU, and DIC) among the population during two peak periods of SARS-CoV-2 infections and two periods of seasonal respiratory infections in Cluj-Napoca, Romania, using the WBE approach.

For this purpose, specific and sensitive methods such as solid phase extraction and liquid chromatography coupled with tandem MS/MS have been developed to allow the analysis of selected compounds at trace levels present in wastewater.

The use of the WBE methodology allowed the provision of information related to the consumption of selected pharmaceuticals, respectively, and an overview of the consumption behavior of the population during sensitive periods from a public health point of view.

The great advantage of WBE is that it can provide information on the consumption of pharmaceuticals, especially if they are not recorded in official databases, since consumption is measured by human metabolic excretion products (biomarkers) in wastewater, as in the case of our study. In addition, further investigations should be carried out into the basic compounds considered biomarkers in wastewater, given that they originate from both human consumption, veterinary use, and expired pharmaceuticals discharged into the sewage system.

The biggest challenge of this study was related to estimating the number of inhabitants connected to the sewage system, as this parameter can be influenced by events, industrial and domestic discharges, or weather.

Among the hydrochemical parameters considered for estimating the population connected to the sewage system, NH_4_-N was chosen because it is specific to urinary excretion, without being affected by industrial or household activities, and is affected only by the rainy season.

The results obtained in the present study showed consumption values comparable to values obtained in other worldwide studies, with the mention that an increasing trend is observed in the case of IBU, DIC, and KET. The highest values for PARA and NSAIDs were obtained during the peak periods of the COVID-19 pandemic. This consumption can be attributed to the pandemic, but errors associated with the results and deriving from a wrong estimate of the number of inhabitants, respectively, and meteorological events during the monitoring periods, should not be excluded.

An increased incidence of PARA and NSAIDs in wastewater was expected due to their frequent use during the pandemic and their availability without a medical prescription. PARA and IBU are also recommended as medications to relieve symptoms following vaccination, fever, and flu pain, and other NSAIDs are used in various diseases due to their anti-inflammatory properties.

Finally, we believe that the present study provides very important information regarding the applicability and limits of WBE in estimating pharmaceutical consumption and can be used as a starting point in establishing more efficient pharmaceutical consumption monitoring programs.

## Figures and Tables

**Table 1:** The minimum and maximum values of selected compounds for monitoring campaigns.

Compound	Concentration (μg/L)							
	September 2021		February 2022		February 2024		October 2024	
	Min.	Max.	Min.	Max.	Min.	Max.	Min.	Max.
PARA	6.46	24.83	0.88	12.14	2.20	9.82	4.57	8.27
KET	0.90	9.34	0.75	3.66	2.45	3.09	3.34	3.98
NAP	1.28	1.92	1.44	2.62	1.96	3.45	2.78	3.75
IBU	2.50	5.06	1.33	2.52	5.52	7.93	8.79	11.80
DIC	0.97	3.04	1.12	2.36	3.02	4.73	2.67	4.21

**Table 2:** The minimum, maximum, and average values of the consumption of the selected compounds for monitoring campaigns.

Compound	Consumption (g/d/1000 inh)											
	September 2021			February 2022			February 2024			October 2024		
	Min.	Max.	Average	Min.	Max.	Average	Min.	Max.	Average	Min.	Max.	Average
PARA	44.60	185.57	104.19	6.65	93.46	34.70	15.17	69.48	51.29	29.36	54.28	45.45
KET	0.25	2.65	1.03	0.21	0.99	0.56	0.66	0.79	0.75	0.81	0.98	0.89
NAP	0.29	0.46	0.39	0.35	0.60	0.44	0.44	0.82	0.64	0.57	0.78	0.68
IBU	0.55	1.15	0.81	0.32	0.61	0.43	1.25	1.83	1.61	1.76	2.44	1.92
DIC	0.23	0.73	0.43	0.27	0.57	0.36	0.72	1.11	0.87	0.54	0.87	0.72

**Table 3:** Estimated consumption (g/d/1000 inh) of PARA and NSAIDs in different countries using WBE.

Country	WWTP	PARA	KET	NAP	IBU	DIC	Ref.
Austria	Innsbruck	1.80–15.20	–	–	–	–	[14]
		3.70–14.00	–	–	–	–	
Greece	Athens	19.01	0.11	10.34	0.75	0.63	[28]
		56.61	0.28	10.97	1.88	1.00	
United Kingdom	Keynsham	61.29 ± 48.13	0.14 ± 0.17	4.16 ± 3.99	3.79 ± 2.12	0.13 ± 0.09	[32]
	Bath	47.89 ± 18.14	0.14 ± 0.11	3.56 ± 2.67	3.29 ± 2.16	0.14 ± 0.05	
Vietnam	Ho Chi Minh (A)	473.0 ± 131.0	–	–	4.60 ± 0.50	–	[60]
	Ho Chi Minh (B)	283.0 ± 0.05	–	–	3.60 ± 0.60	–	
South Africa	South Africa (1)	0.79–9.96	–	0.04–0.14	–	0.02–0.094	[61]
	South Africa (2)	1.00–21.37	–	0.19–0.71	–	0.03–0.27	
China	Urban	0.37–0.77	–	2 × 10^−4^–0.51	–	0.04–0.23	[62]
	Rural	0.03–0.84	–	0.23–0.50	–	0.05–0.10	
China	Urban	0.0073–250.0	–	–	0.58–321.0	–	[63]
	Rural	0.112–180.0	–	–	0.38–154.0	–	
Spain	Madrid, Reus, Tarragona	5.91–17.99	–	–	–	0.07–0.74	[64]
United Kingdom	Radstock, Paulton, Bath, Bristol	28.09 ± 11.04–64.24 ± 16.66	0.12–0.15	2.11 ± 0.54–5.41 ± 1.33	3.34 ± 1.17–5.58 ± 1.96	0.10 ± 0.05–0.20 ± 0.10	[65]
Romania	Cluj-Napoca	6.65–185.57	0.21–2.65	0.29–0.82	0.32–2.44	0.23–1.11	This study

“–” not analyzed.

## Data Availability

Data are contained within the article and Supplementary Materials.

## Supplementary Materials

The following supporting information can be downloaded at: https://www.mdpi.com/article/10.3390/metabo15090576/s1, Table S1: The molecular structure and some physico-chemical properties of the studied pharmaceuticals; Table S2: Optimized collision energies, and ions (m/z) of the targeted pharmaceuticals, the retention time (RT), linearity, and limit of detection and quantification of the instrument (LOD, LOQ) and, respectively, of the method (LDM, LQM), as well as the matrix effect; Table S3: The flow rates at the inlet of the wastewater treatment plant (L/s), the concentrations of COD, BOD, P and NH_4_-N (mg/L) provided by the wastewater treatment plant of Cluj-Napoca, as well as the population calculated based on the concentration of hydrochemical parameters for February 2024 and October 2024; Table S4: The concentrations (μg/L) of PARA and studied NSAIDs in the wastewater samples; Table S5: The consumption of PARA and studied NSAIDs estimated among the population using wastewater-based epidemiology.

## Abbreviations

The following abbreviations are used in this manuscript:

WBE	Wastewater-Based Epidemiology
SARS-CoV-2	Severe Acute Respiratory Syndrome Coronavirus 2
COVID-19	Coronavirus Disease 2019
EMA	European Medicines Agency
NSAIDs	Nonsteroidal Anti-Inflammatory Drugs
USD	United States Dollar
LC-MS/MS	Liquid Chromatography–Tandem Mass Spectrometry
d	Day
inh	Inhabitants
IBU	Ibuprofen
KET	Ketoprofen
NAP	Naproxen
DIC	Diclofenac
FA	Formic Acid
PVDF	Polyvinylidene Fluoride
σ	Response Factor of the Detector
S	The Slope of each Calibration Curve
LOD	Limit of Detection
LOQ	Limit of Quantitation
ACN	Acetonitrile
MeOH	Methanol
WWTP	Wastewater Treatment Plant
COD	Chemical Oxygen Demand
BOD	Biological Oxygen Demand
N	Total Nitrogen
NH_4_-N	Nitrogen Ammonia
P	Total Phosphorous
