# Supplementary material for: A Surveillance of Paracetamol and Nonsteroidal Anti-Inflammatory Drug Consumption in Cluj-Napoca, Romania, Using Wastewater-Based Epidemiology
Source: Metabolites. 2025 Aug 28;15(9):576. doi: 10.3390/metabo15090576 (PMC12471657; doi:10.3390/metabo15090576)
Supplement: Supplementary file 1 [file metabolites-15-00576-s001.zip › metabolites-3800970-supplementary.pdf]

SUPPLEMENTARY INFORMATION

Article

A surveillance of paracetamol and nonsteroidal anti-inflammatory drug consumption in Cluj-Napoca, Romania using wastewater-based epidemiology

Mihail Simion Beldean-Galea[1,2*], Mihaela-Cătălina Herghelegiu[1,2*], Audrey Combès[3], Jérome Vial[3], Robert Tötös[4], Maria Concetta Bruzzoniti[5], Maria-Virginia Coman[2]

1 Babeş-Bolyai University, Faculty of Environmental Science and Engineering, 30 Fântânele Str. 30, 400294 Cluj-Napoca, Romania;
2 Babeş-Bolyai University, Raluca Ripan Institute for Research in Chemistry, 30 Fântânele Str. 30, 400294 Cluj-Napoca, Romania;
3 École Supérieure de Physique et de Chimie Industrielle de la Ville de Paris, Paris Sciences et Lettres University, 10 Rue Vauquelin, 75231 Paris Cedex 05, France
4 Babeş-Bolyai University, Faculty of Chemistry and Chemical Engineering, Árány Janos Str. 11, 400028 Cluj-Napoca, Romania
5 University of Turin, Department of Chemistry, via P. Giuria 5, 10125, Turin, Italy
* Correspondence: *simion.beldean@ubbcluj.ro* (M.S.B.G.), *mihaela.herghelegiu@ubbcluj.ro* (M.C.H.)

The supplementary information contains 9 pages, and includes 5 tables and 0 figures.

List of contents of the supplementary information:

Table S1. The molecular structure and some physico-chemical properties of the studied pharmaceuticals.

Table S2. Optimized collision energies, and ions (*m/z*) of the targeted pharmaceuticals, the retention time (RT), linearity, and limit of detection and quantification of the instrument (LOD, LOQ) and, respectively, of the method (LDM, LQM), as well as the matrix effect.

Table S3. The flow rates at the inlet of the wastewater treatment plant (L/s), the concentrations of COD, BOD, P and NH_4-N (mg/L) provided by the wastewater treatment plant of Cluj-Napoca, as well as the population calculated based on the concentration of hydrochemical parameters for February 2024 and October 2024.

Table S4. The concentrations (μg/L) of PARA and studied NSAIDs in the wastewater samples.

Table S5. The consumption of PARA and studied NSAIDs estimated among the population using wastewater-based epidemiology.

Table S1. The molecular structure and some physico-chemical properties of the studied pharmaceuticals.

Pharmaceu-ticals	Molecular structure	Chemical formula	Molecular mass (amu)	LogP	pKa
**Paracetamol**	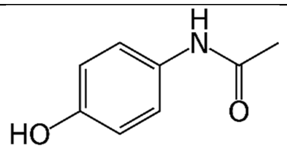	$C_8H_9NO_2$	151.16	0.91	9.38
**Ketoprofen**	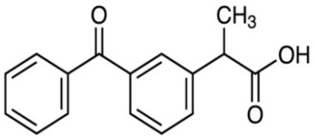	$C_{16}H_{14}O_3$	254.28	3.12	4.45
**Naproxen**	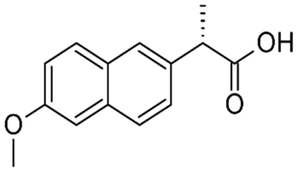	$C_{14}H_{14}O_3$	230.26	3.18	4.15
**Ibuprofen**	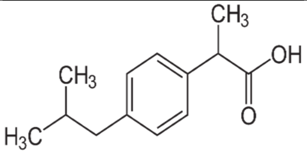	$C_{13}H_{18}O_2$	206.28	3.97	4.91
**Diclofenac**	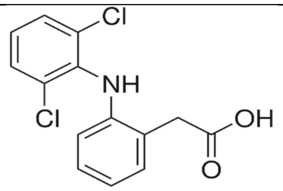	$C_{14}H_{11}Cl_2NO_2$	296.14	4.51	4.15

Physicochemical properties (Molecular mass, LogP, pKa) from PubChem databases. https://pubchem.ncbi.nlm.nih.gov/ (accessed on July 2025)

Table S2. Optimized collision energies, and ions (*m/z*) of the targeted pharmaceuticals, the retention time (RT), linearity, and limit of detection and quantification of the instrument (LOD, LOQ) and, respectively, of the method (LDM, LQM), as well as the matrix effect.

Pharma-ceuticals	RT (min)	Precursor Ion (*m/z*)	Product Ions (*m/z*)	Collision Energy (V)	Calibration curve equation	SD	R^2	LOD (µg/L)	LOQ (µg/L)	LDM (ng/L)	LQM (ng/L)	Precision (RSD%)		Matrix effect (%)
												Intra-day	Inter-day	
**PARA**	3.50	152.1	110.1	16	y = 57,897,563.37x+4,288,501.6	919,455.66	0.9847	52	158	52	158	14.05	7.58	70–186
			93.1	23										
**KET**	9.65	252.9	208.0	–10	y = 7,664.3x-1,172.6	77.65	0.9844	30	101	30	101	4.71	4.72	5–91
			197.4	–9										
**NAP**	10.08	229.0	185.0	–10	y = 1,273,701.28x+45,920.17	2,344.29	0.9993	6	18.4	6	18.4	4.02	5.78	16–46
			169.0	–40										
**IBU**	11.33	205.1	159.2	–10	y = 1,072,384.60x+12,926.82	16,762.74	0.9977	51	156	51	156	4.30	5.78	21–70
			161.0	–10										
**DIC**	11.57	293.8	249.9	–15	y = 27,178,096.20x+646,587.26	49,485.26	0.9981	6	18.2	6	18.2	2.54	4.11	18–71
			214.1	–23										

Table S3. The flow rates at the inlet of the wastewater treatment plant (L/s), the concentrations of COD, BOD, P and NH_4-N (mg/L) provided by the wastewater treatment plant of Cluj-Napoca, as well as the population calculated based on the concentration of hydrochemical parameters for February 2024 and October 2024.

Day	Flow (L/s)	COD (mg/L)	Population based on COD	BOD (mg/L)	Population based on BOD	P (mg/L)	Population based on P	NH_4-N (mg/L)	Population based on NH_4-N
				February 2024					
**08.02.2024**	1,079.00	395.50	288,053	–	–	–	–	–	–
**09.02.2024**	1,128.00	389.50	296,565	–	–	–	–	–	–
**10.02.2024**	1,132.00	216.50	165,428	–	–	–	–	–	–
**11.02.2024**	1,149.00	308.20	239,032	–	–	–	–	–	–
**12.02.2024**	1,036.00	346.20	242,098	144.20	215,123	6.35	334,348	41.71	439,233
**13.02.2024**	1,093.00	636.00	469,225	246.90	388,601	–	–	–	–
**14.02.2024**	1,093.00	365.40	269,583	–	–	–	–	–	–
**Average**			**281,426**		**301,862**		**334,348**		**439,233**
				October 2024					
**21.10.2024**	1,212.96	445.60	364,835	214.40	374,485	6.84	421,666	43.17	532,261
**22.10.2024**	1,187.50	370.00	296,578	185.60	317,376	–	–	–	–
**23.10.2024**	1,192.13	350.20	281,802	–	–	–	–	–	–
**24.10.2024**	1,164.35	351.60	276,336	–	–	–	–	–	–
**25.10.2024**	1,215.28	363.90	298,512	–	–	–	–	–	–
**26.10.2024**	1,157.41	330.00	257,813	–	–	–	–	–	–
**27.10.2024**	1,187.50	282.90	226,762	–	–	–	–	–	–
**Average**			**286,091**		**345,931**		**421,666**		**532,261**

Table S4. The concentrations (µg/L) of PARA and NSAIDs studied in the wastewater samples.

**Day**	**Concentrations (µg/L)**				
	**PARA**	**KET**	**NAP**	**IBU**	**DIC**
September 2021					
**07.09.2021**	24.83	5.46	1.49	3.36	1.94
**08.09.2021**	14.09	1.32	1.62	2.66	0.97
**09.09.2021**	10.67	9.34	1.92	3.09	2.05
**10.09.2021**	14.56	5.24	1.85	5.06	1.92
**11.09.2021**	15.44	2.47	1.28	3.46	1.92
**12.09.2021**	11.90	0.90	1.67	3.28	1.62
**13.09.2021**	13.35	2.61	1.81	3.50	3.04
**14.09.2021**	6.46	1.28	1.68	2.50	1.11
**21.09.2021**	15.59	4.58	1.89	4.32	2.00
**Average**	**14.19**	**3.82**	**1.68**	**3.47**	**1.84**
February 2022					
**08.02.2022**	2.64	3.66	2.62	1.79	1.35
**09.02.2022**	12.14	1.73	1.70	2.52	2.36
**10.02.2022**	5.10	3.31	1.77	1.33	1.68
**11.02.2022**	5.85	2.27	1.76	2.40	1.31
**12.02.2022**	3.78	1.41	1.52	1.78	1.50
**13.02.2022**	4.42	0.93	2.00	1.38	1.41
**14.02.2022**	3.85	1.76	1.44	1.45	1.42
**15.02.2022**	0.88	0.75	1.78	1.56	1.12
**16.02.2022**	2.48	1.99	1.97	2.16	1.33
**Average**	**4.57**	**1.98**	**1.84**	**1.82**	**1.50**
February 2024					
**08.02.2024**	9.82	2.77	2.90	7.93	4.09
**09.02.2024**	7.64	2.71	2.66	7.53	3.45
**10.02.2024**	7.41	2.80	2.88	7.84	4.73
**11.02.2024**	7.53	2.75	3.45	7.44	3.02
**12.02.2024**	6.29	3.09	3.03	6.98	4.30
**13.02.2024**	2.20	2.88	1.96	5.52	3.45
**14.02.2024**	8.77	2.45	2.70	6.27	3.74
**Average**	**7.09**	**2.78**	**2.80**	**7.07**	**3.83**

		October 2024			
**21.10.2024**	8.27	3.97	3.75	11.80	4.21
**22.10.2024**	7.62	3.78	3.21	9.49	3.83
**23.10.2024**	8.17	3.65	3.64	9.12	3.47
**24.10.2024**	7.31	3.76	3.34	8.89	3.89
**25.10.2024**	6.94	3.69	2.78	9.11	3.07
**26.10.2024**	6.57	3.62	2.98	8.99	3.98
**27.10.2024**	4.57	3.34	3.66	8.79	2.67
**Average**	**7.06**	**3.69**	**3.34**	**9.45**	**3.59**

Table S5. The consumption of PARA and studied NSAIDs estimated among the population using wastewater-based epidemiology.

Day	Estimated consumption (g/d/1000inh)				
	**PARA**	**KET**	**NAP**	**IBU**	**DIC**
	September 2021				
**07.09.2021**	185.57	1.53	0.35	0.79	0.46
**08.09.2021**	104.88	0.37	0.38	0.63	0.23
**09.09.2021**	80.81	2.65	0.46	0.74	0.49
**10.09.2021**	104.79	1.41	0.42	1.15	0.44
**11.09.2021**	110.83	0.66	0.29	0.78	0.44
**12.09.2021**	87.32	0.25	0.39	0.76	0.37
**13.09.2021**	101.41	0.74	0.43	0.84	0.73
**14.09.2021**	44.60	0.33	0.37	0.55	0.24
**21.09.2021**	117.47	1.29	0.45	1.03	0.48
**Average**	**104.19**	**1.03**	**0.39**	**0.81**	**0.43**
	February 2022				
**08.02.2022**	19.03	0.99	0.60	0.41	0.31
**09.02.2022**	93.46	0.50	0.41	0.61	0.57
**10.02.2022**	39.27	0.95	0.43	0.32	0.41
**11.02.2022**	42.76	0.62	0.41	0.55	0.30
**12.02.2022**	29.53	0.41	0.38	0.44	0.37
**13.02.2022**	33.73	0.27	0.48	0.33	0.34
**14.02.2022**	29.38	0.50	0.35	0.35	0.34
**15.02.2022**	6.65	0.21	0.43	0.37	0.27
**16.02.2022**	18.52	0.56	0.46	0.51	0.31
**Average**	**34.70**	**0.56**	**0.44**	**0.43**	**0.36**
	February 2024				
**08.02.2024**	69.48	0.73	0.65	1.77	0.91
**09.02.2024**	56.51	0.75	0.62	1.75	0.80
**10.02.2024**	55.00	0.78	0.67	1.83	1.11
**11.02.2024**	56.73	0.78	0.82	1.77	0.72
**12.02.2024**	42.73	0.79	0.65	1.49	0.92

**13.02.2024**	15.77	0.77	0.44	1.25	0.78
**14.02.2024**	62.85	0.66	0.61	1.42	0.84
**Average**	**51.29**	**0.75**	**0.64**	**1.61**	**0.87**
			October 2024		
**21.10.2024**	54.28	0.98	0.78	2.44	0.87
**22.10.2024**	48.96	0.91	0.65	1.92	0.77
**23.10.2024**	52.70	0.88	0.74	1.85	0.70
**24.10.2024**	46.05	0.89	0.66	1.76	0.77
**25.10.2024**	45.64	0.91	0.57	1.89	0.64
**26.10.2024**	41.15	0.85	0.59	1.77	0.78
**27.10.2024**	29.36	0.81	0.74	1.78	0.54
**Average**	**45.45**	**0.89**	**0.68**	**1.92**	**0.73**
